# Differential Regulation of Male-Hormones-Related Enhancers Revealed by Chromatin Accessibility and Transcriptional Profiles in Pig Liver

**DOI:** 10.3390/biom14040427

**Published:** 2024-04-01

**Authors:** Shuheng Chan, Yubei Wang, Yabiao Luo, Meili Zheng, Fuyin Xie, Mingming Xue, Xiaoyang Yang, Pengxiang Xue, Chengwan Zha, Meiying Fang

**Affiliations:** 1 Department of Animal Genetics and Breeding, National Engineering Laboratory for Animal Breeding, MOA Key Laboratory of Animal Genetics and Breeding, Beijing Key Laboratory for Animal Genetic Improvement, State Key Laboratory of Animal Biotech Breeding, Frontiers Science Center for Molecular Design Breeding, College of Animal Science and Technology, China Agricultural University, Beijing 100193, China; shubert@cau.edu.cn (S.C.); yabiaoluo@cau.edu.cn (Y.L.); pxxue@cau.edu.cn (P.X.);; 2 Sanya Institute of China Agricultural University, Sanya 572025, China; 3 Beijing General Station of Animal Husbandry, Beijing 100107, China

**Keywords:** surgical castration, regulatory factors, *HSD3B1*, androstenone metabolism, *SP1*, enhancer, andEN, pork quality

## Abstract

Surgical castration can effectively avoid boar taint and improve pork quality by removing the synthesis of androstenone in the testis, thereby reducing its deposition in adipose tissue. The expression of genes involved in testis-derived hormone metabolism was altered following surgical castration, but the upstream regulatory factors and underlying mechanism remain unclear. In this study, we systematically profiled chromatin accessibility and transcriptional dynamics in liver tissue of castrated and intact full-sibling Yorkshire pigs. First, we identified 897 differentially expressed genes and 6864 differential accessible regions (DARs) using RNA- and ATAC-seq. By integrating the RNA- and ATAC-seq results, 227 genes were identified, and a significant positive correlation was revealed between differential gene expression and the ATAC-seq signal. We constructed a transcription factor regulatory network after motif analysis of DARs and identified a candidate transcription factor (TF) *SP1* that targeted the *HSD3B1* gene, which was responsible for the metabolism of androstenone. Subsequently, we annotated DARs by incorporating H3K27ac ChIP-seq data, marking 2234 typical enhancers and 245 super enhancers involved in the regulation of all testis-derived hormones. Among these, four typical enhancers associated with *HSD3B1* were identified. Furthermore, an in-depth investigation was conducted on the androstenone-related enhancers, and an androstenone-related mutation was identified in a newfound candidatetypical enhancer (andEN) with dual-luciferase assays. These findings provide further insights into how enhancers function as links between phenotypic and non-coding area variations. The discovery of upstream TF and enhancers of *HSD3B1* contributes to understanding the regulatory networks of androstenone metabolism and provides an important foundation for improving pork quality.

## 1. Introduction

The growing world population is driving up the need for food on a daily basis. High-quality, mass-produced meat is required, as it is the most accessible and simplest kind of protein. Studies have indicated that since there is less fat accumulation on intact (un-castrated) males, they develop quicker and have better feed efficiency [1,2]. Breeding intact males is also desirable to avoid pain during surgical castration but raises issues on meat quality, particularly on boar taint. Boar taint is a meat quality issue characterized by an off-odor or off-flavor in pork products, which is primarily caused by the accumulation of androstenone (5α-androst-16-en-3-one) and skatole in the adipose tissue [3,4,5]. Androstenone is one of the main steroid hormones in the testis and mainly degrades in the liver, and it is subsequently delivered and deposited in adipose tissue because of its lipophilic properties [6,7,8]. Traditionally, male pigs are routinely castrated shortly after birth to prevent boar taint by removing the synthesis of androstenone in the testis, thereby reducing its deposition in adipose tissue. However, castration is not the best solution due to ethical and economical concerns. Compared with castrated pigs, intact male pigs grow faster and have improved feed efficiency owing to reduced fat deposition [2,9]. In addition, castration is associated with a high risk of infection and complications [10]. Therefore, decreasing the synthesis of androstenone in the testis, such as by castration, or improving the metabolism of androstenone in the liver can effectively reduce boar taint. Several studies have found that the differentially expressed genes in castrated and non-castrated boars’ livers are related to androstenone metabolism [11,12], but the mechanism behind it remains unclear.

Androstenone levels are predominantly genetically determined, which have relatively high heritability estimates ranging from 0.25 to 0.88 and vary greatly depending on the breed [13,14]. Over the past two decades, numerous studies based on microsatellite and single nucleotide polymorphism (SNP) markers have identified several quantitative trait locus (QTL) regions for androstenone, located on multiple chromosomes (SSC 1, 2, 3, 5, 6, 7, 9, 13, 14, and 15), and several candidate genes have been identified, such as a subclass of *HSD*, *CYP*, and *SULT* genes [15,16,17,18,19]. Furthermore, differential expression analysis revealed a series of candidate genes involved in androstenone synthesis and metabolism [11,20,21,22,23,24,25]. A linkage study using genotype data from the 80K SNP panel and RNA-seq data from Danish cross-bred pigs reported 262 expression quantitative trait Loci (eQTLs) associated with androstenone in pig testis [25], further promoting our understanding of androstenone biosynthesis. The metabolism of androstenone occurs in two phases: Phase I involves hydrogenation metabolism [8,26,27,28,29], while Phase II involves sulfoconjugation metabolism in the liver or testis [30,31,32,33]. There is also evidence that glucuronidation metabolism is another metabolic pathway of androstenone [26,28,34]. Researchers have dedicated decades to investigating the enzymes involved in the metabolism of androstenone, yielding a comprehensive understanding of key genes, including *HSD3B1* and *SULT2A1* [8,26,30], but the upstream regulatory factors such as enhancers and underlying mechanism remain unclear.

Gene expression programs are orchestrated by specific transcription factors (TFs) that bind to cis-regulatory elements [35]. The genome-wide profiling of TF binding events and regulatory elements has contributed to our understanding of biological processes. In eukaryotes, genomic DNA is wrapped around histones to form nucleosomes, which are assembled into chromatin [36]. The composition and post-translational modifications of nucleosomes reflect different functional states and regulate chromatin accessibility via multiple mechanisms [37,38]. The degree of physical contact between chromatinized DNA and nuclear macromolecules, such as TFs, is known as chromatin accessibility [36]. The arrangement of chromatin accessibility reflects the dynamic regulation of gene expression via interactions with TFs and cofactors.

The assay for transposase-accessible chromatin with high-throughput sequencing (ATAC-seq) is a robust method for defining regions of accessible chromatin based on Tn5 transposase activity. ATAC-seq has been extensively utilized to determine cis-regulatory elements and predict TF binding sites [39]. Thus far, most studies on androstenone have focused only on genomic sequencing or gene expression profiling.

In this study, we systematically profiled chromatin accessibility and transcriptional dynamics in liver tissue of castrated and intact full-sibling Yorkshire pigs. By integrating RNA-, ATAC-, and ChIP-seq data, we identified upstream regulatory factors, including TF *SP1* and four enhancers of *HSD3B1*. The results of our work reveal the gene expressions and underlying mechanisms associated with chromatin accessibility and TF binding and provide a novel layer of epigenetic regulation of gene expression in pig liver.

## 2. Materials and Methods

### 2.1. Animals and Sample Collection

In this study, six full-sibling male Yorkshire piglets were divided into three pairs according to the principle of pairing design: each pair of male piglets comprised two full siblings from the same litter with identical body weights, and the initial conditions of the test individuals were maintained. At one week of age, one piglet was randomly selected from each pair for surgical castration. Conversely, the other piglet was not castrated and received the sham treatment, which was an incision of the same size as the castration operation but made in the abdomen on the premise of not damaging the gonads to produce the same stress effect, and that piglet functioned as the control. All pigs were bred under the same conditions, received free access to water and feed, and were slaughtered at 180 days of age. The Yorkshire pigs were raised by the Beijing Zhongyu Pig Co., Ltd. (Beijing, China).

### 2.2. ATAC-Seq

Libraries for the ATAC-seq were generated using six samples (Castrated pigs = 3, Intact pigs = 3). The nuclei were extracted and then incubated with 50 µL of Tn5 transposition reaction mix at 37 °C for 30 min, and the DNA was purified using the MolPure^®^ (Yeasen, Shanghai, China) PCR Purification Kit [39]. The qualified libraries were sequenced (150-bp paired-end) on Illumina Novaseq 6000 (Allwegene Technology, Beijing, China). Raw sequence reads were initially processed for quality control using FastQC, after which the adapter sequences and poor-quality reads were removed. Quality-filtered reads were then mapped to the reference genome (*Sus scrofa* 11.1) using BWA or Bowtie, and only uniquely mapped reads were retained, converted to Bam format using SAMtools, and used for peak calling. MACS2 was used to call peaks with the sonicated input as a control and an initial threshold q-value of 0.05 as the cutoff. Bedtools was used to merge the peaks to identify differences in accessibility. Peaks of each sample corresponding to the genes were annotated. The HOMER tool suite was used for TF motif discovery by analyzing differential motif enrichment in castration/intact group-specific and shared differential regulatory element datasets against all element (peak) backgrounds.

### 2.3. RNA-Seq

Illumina high-throughput sequencing was used to sequence total RNA from liver samples. The RNA sample preparation process used a total of amount of 2 µg of RNA per sample as the initial substance. Following the manufacturer’s instructions, sequencing libraries were created using the NEBNext^®^ Ultra™ RNA Library Prep Kit for Illumina^®^ (NEB, Ipswich, MA, USA). Original data for each sample were subjected to a quality control assessment using FastQC, and high-quality clean reads were obtained by filtering the adapter and low-quality reads using Trimmomatic (v0.39). Subsequently, they were mapped to the porcine reference genome (*Sus scrofa* 11.1) using HiSAT2 (v2.2.1). Differential expression analyses were performed using the DESeq2 R package. Significantly differentially expressed genes (DEGs) were identified based on the following criteria: |log2(fold change)| ≥ 1 and *p* < 0.05. Three biological replicates were used.

### 2.4. Integration Analysis of ATAC-Seq and RNA-Seq

The ATAC-seq results were combined with the RNA-seq expression results to compare the DEGs in open chromatin regions and key TFs. The upregulated and downregulated DEGs in the RNA-seq analysis were compared with genes associated with differential peaks in ATAC-seq. Gene ontology (GO) and Kyoto Encyclopedia of Genes and Genomes (KEGG) pathway analyses were performed using an online resource (http://www.omicshare.com/) (accessed on 28 September 2023) with default instructions. For these overlapping DEGs, gene set enrichment analyses were conducted using an online resource (http://www.omicshare.com/) (accessed on 28 September 2023) with default instructions. Significantly enriched GO biological processes and KEGG pathways were identified with *p*- and q-value cutoffs of 0.01 and 0.05, respectively.

### 2.5. Enhancer Identification and Analysis

The ChIP-seq signal of H3K27ac from the liver of male Yorkshire piglets of 180 days of age [40,41] was used to define super enhancers (SEs). A set of large enhancer clusters with an exceptionally high density of H3K27ac was defined as SEs, whereas the others were considered typical enhancers (Ens). SEs were identified and annotated using default parameters according to the ROSE algorithm [40,41]. The chromatin states for different tissues and Hi-C data were from UCSC browser (http://genome.ucsc.edu/s/zhypan/susScr11_15_state_14_tissues_new) (accessed on 23 October 2023).

### 2.6. Dual-Luciferase Reporter Assay

The predicted enhancers were confirmed using a dual-luciferase reporter assay system [42]. Enhancer fragments were cloned into the pGL4.23[luc2/minP] vector (Promega, Madison, WI, USA, E8411), and 293T cells (ATCC, Manassas, VA, USA, ACS-4004) were transfected using Lipofectamine 2000 (Invitrogen, Carlsbad, CA, USA) and then incubated for 36 h. The relative luciferase activity (RL/FL) was determined using a Dual-Luciferase^®^ Reporter Assay System (Promega, E1910), according to the manual.

### 2.7. Gene Expression and Statistical Analysis

The expressions of the selected genes were confirmed using RT-qPCR. Total RNA was isolated from the liver samples via TRIzol/chloroform/isopropanol extraction using a standard protocol (Invitrogen, USA). cDNA was generated using FastKing gDNA Dispelling RT SuperMix (Tiangen Biotech, Beijing, China). RT-qPCR was performed in duplicate, and each gene was normalized to the housekeeping gene, GAPDH. Three biological replicates were used. The primers used in this study are listed in Supplementary Table S1. All the data are expressed as means ± SEM. The normality of experimental data was assessed using the Shapiro–Wilk test either on the original data or after log-transformation; differential expression was analyzed using a *t*-test and one-way ANOVA after conforming to a Gaussian distribution, with values of *p* < 0.05 (*) or *p* < 0.01 (**), indicating significance.

## 3. Results

### 3.1. ATAC-Seq Quality Control of the Intact and Castrated Male Pig Liver Tissues

**Figure 1:**
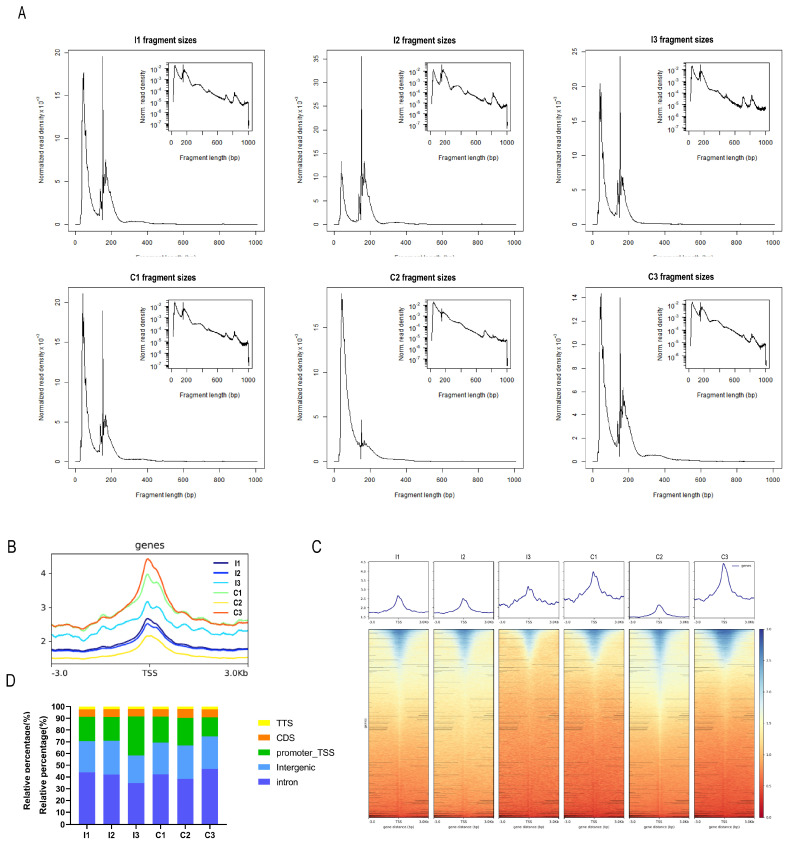
ATAC−seq quality control. (**A**) Frequency distribution of fragment lengths within a representative ATAC−seq library. The smallest fragment peaks represent sequencing reads in inter-nucleosome open chromatin, while larger peaks represent those spanning nucleosomes. (**B**) Mapped read distributions across gene bodies and peaks. (**C**) Enrichment of ATAC−seq signals within a 3 kb region upstream and downstream of the transcription start site (TSS) in each sample. (**D**) Genomic distribution of the peaks in each sample.

To investigate the chromatin accessibility dynamics in the liver tissue of castrated and intact full-sibling Yorkshire pigs, we performed ATAC-seq in the liver tissue isolated from the intact (I group) and castrated (C group) pigs. A total of 590,776,030 raw reads were obtained. On average, >82 million high-quality clean reads were acquired per sample following filtration (Supplementary Table S2). All the libraries yielded fragment lengths with the expected distribution, indicating good data quality. The highest peak on the left was the nucleosome-free fragment corresponding to the open chromatin region. The mononucleosome peak, a cleavage fragment including a nucleosome greater than 147 and less than 147 × 2, also appeared in the length distribution map (Figure 1A), which was similar to previously reported ATAC-seq data [43]. Most of the identified accessible areas were enriched within 3 kb of the transcription start site (TSS) (Figure 1B), indicating that the regions of open chromatin (ROCs) participated in transcriptional regulation. A heatmap reflected the enrichment of reads in the regions 3 kb upstream and downstream of the TSS of all genes in the genome (Figure 1C). These two groups had different signal intensities, suggesting that several factors may be involved in their regulation. Most peaks across all samples were mapped to the intronic, intergenic, and promoter regions (Figure 1D), which is consistent with similar studies in pigs [44]. These results suggest the reliability and good reproducibility of our ATAC-seq data.

### 3.2. Genome-Wide Identification of Accessible Chromatin Regions

**Figure 2:**
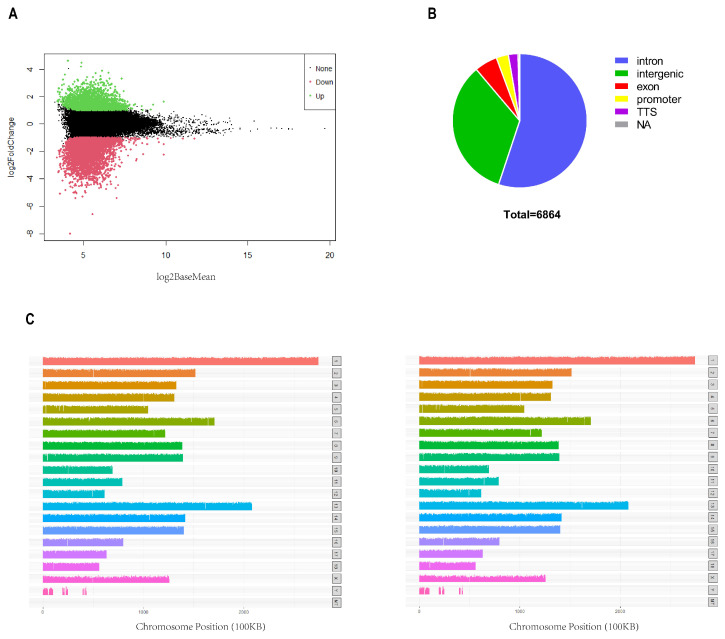
Characterization of DARs. (**A**) MA plot, where the x−axis represents signal strength and y-axis represents log2 (fold change). (**B**) Genomic distribution of differential peaks. (**C**) Distribution of differential peaks on the chromosomes (left: intact group, right: castration group).

We identified 102,384 and 94,479 accessible chromatin peaks in the C and I groups, respectively. Subsequently, 54,545 and 6864 common and differential peaks were screened, respectively (Figure 2A). We annotated the genomic distribution of differential peaks using the annotation file, and the majority of peaks were mapped to intronic (54.45%) and intergenic (33.06%) regions, as expected (Figure 2B). These regions primarily contain cis-regulatory elements that are typically positively correlated with gene transcription. The distribution map of all the peaks on the chromosomes is shown in Figure 2C. Most regions of each chromosome were covered, while certain chromosomes, such as ChrY, were less covered.

### 3.3. RNA-Seq Data from the Intact and Castrated Male Pig Liver Tissues

**Figure 3:**
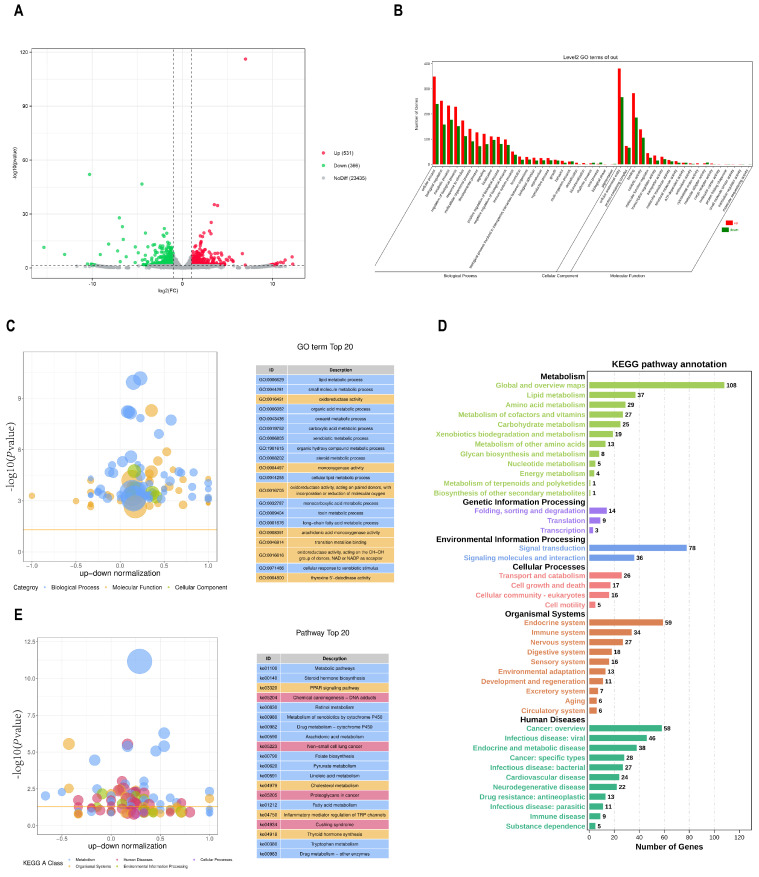
RNA−seq analysis. (**A**) Volcano plot of the transcriptome data. (**B**) GO annotation of DEGs. (**C**) Top 20 GO terms of GO enrichment analysis. (**D**) KEGG pathway annotation of DEGs. (**E**) Top 20 pathways of KEGG enrichment analysis. (Significantly enriched pathways are indicated above the yellow line (*p* < 0.05)).

To investigate the link between chromatin accessibility and gene expression, we performed RNA-seq on the same ATAC-seq sample. A total of 534 million reads were obtained from transcriptome sequencing of the six samples. On average, >81 million high-quality clean reads were obtained per sample following filtration (Supplementary Table S3). To identify key functional genes related to the phenotype, DEGs were filtered based on the criteria |log2(fold change)| ≥ 1 and *p* < 0.01. Overall, 897 DEGs were identified, including 531 upregulated and 366 downregulated genes (Figure 3A). GO and KEGG pathway enrichment analyses were used to decipher the function of these DEGs. The results showed that the biological processes of GO analysis were primarily enriched in cellular process, biological regulation, and metabolic process. Cellular components were primarily enriched in cellular anatomical and protein-containing complex, and molecular functions were primarily enriched in binding and catalytic activity (Figure 3B). Notably, almost all the top 20 GO terms were related to metabolism (Figure 3C). In addition, several metabolism-associated KEGG pathways, including lipid metabolism, folding, sorting, degradation, signal transduction, transport, and catabolism, were annotated (Figure 3D), and the top 20 pathways are shown in Figure 3E, including metabolic pathways (ko01100) and cytochrome P450 (ko00982). This suggests that the DEGs may be involved in testis-derived steroid hormones metabolism.

### 3.4. Integration of the ATAC- and RNA-Seq Results

**Figure 4:**
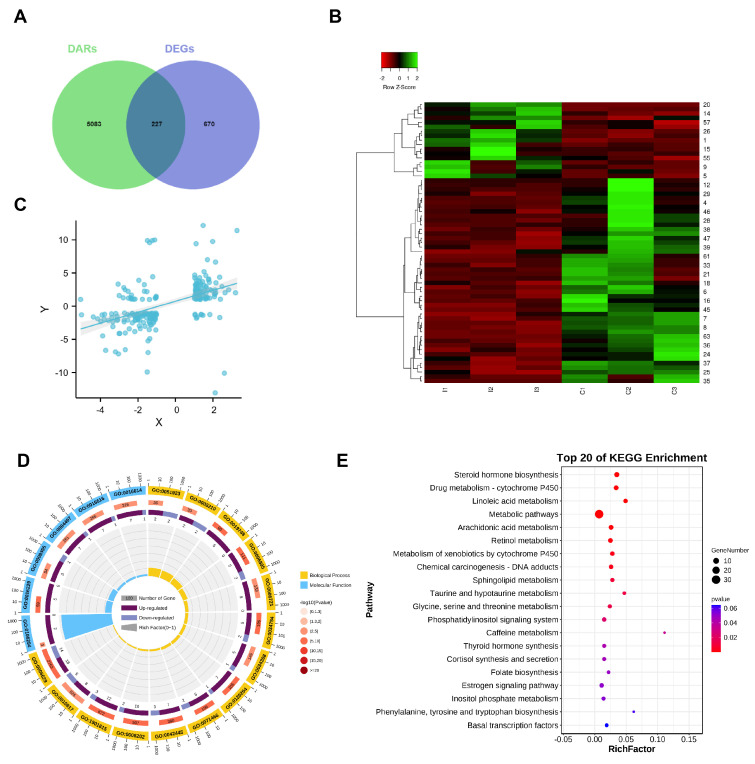
Integrated analysis of ATAC− and RNA−seq data. (**A**) Overlap of DEGs identified by ATAC− and RNA−seq. (**B**) Heatmap of the overlapping genes. (**C**) Correlation of significantly differentially accessible genes (ATAC−seq) and gene expressions (RNA−seq). R = 0.559, *p* < 0.001. (**D**) GO enrichment of the overlapping genes. (**E**) Top 20 significantly enriched KEGG pathways.

To further determine the relationship between chromatin accessibility and gene expression, an integrated analysis of the ATAC- and RNA-seq datasets was conducted to examine the potential correlations between alterations in open chromatin regions and gene expression levels. A total of 227 overlapping genes were identified (Figure 4A), and heat maps of these overlapping genes are shown in Figure 4B. We assumed that chromatin accessibility and transcription were functionally tied together, which was supported by the significant positive correlation between chromatin accessibility and the RNA expressions of these 227 overlapping genes (Figure 4C). As shown in Supplementary Table S4, 87 genes were upregulated in the region with higher chromatin accessibility, and 99 genes were downregulated in the region with lower chromatin accessibility. In contrast, only 11 genes were downregulated in the region with higher chromatin accessibility, and 30 genes were upregulated in the region with lower chromatin accessibility. This may be due to the involvement of transcription factors or repressors in regulating gene expression.

The top 20 GO terms according to the GO annotation of these overlapping genes are shown in Figure 4D. Among them, as a steroid, androstenone-associated biological processes, including steroid metabolic processes (GO:0008202), molecular function categories of steroid dehydrogenase activity (GO:0016229), which are involved in the conversion to the hydroxyl form of androstenone, and oxidoreductase activity (GO:0016616, GO:0016614) were significantly enriched. Moreover, DEGs were significantly associated with steroid hormone biosynthesis (ko00140) and the drug metabolism-cytochrome P450 (ko00982) (Figure 4E). This suggested that those genes may be involved in androstenone metabolism.

**Figure 5:**
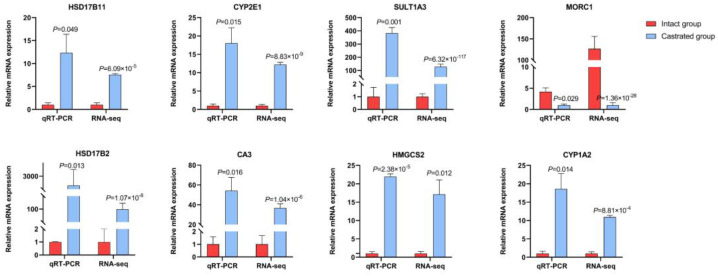
Confirmation of RNA−seq data using qRT−PCR. The relative expression determined by qRT−PCR are shown on the left, and the FPKM determined by RNA−seq are shown on the right. All data represent the average value of three biological replicates with standard errors, and all data are normalized.

To confirm the accuracy of the RNA-seq data, eight genes randomly selected from the 227 DEGs were analyzed using qRT-PCR, including genes of the dehydrogenase family and the cytochrome P450 family. The results showed that the expression patterns of these genes were consistent with the trend observed in the transcriptomic data (Figure 5), which confirmed that DEGs identified from RNA-seq in this study were reliable. Therefore, DEGs identified by ATAC- and RNA-seq should be further investigated as candidate genes.

### 3.5. Corresponding Genes and Motif Analysis in Differential Peaks

**Figure 6:**
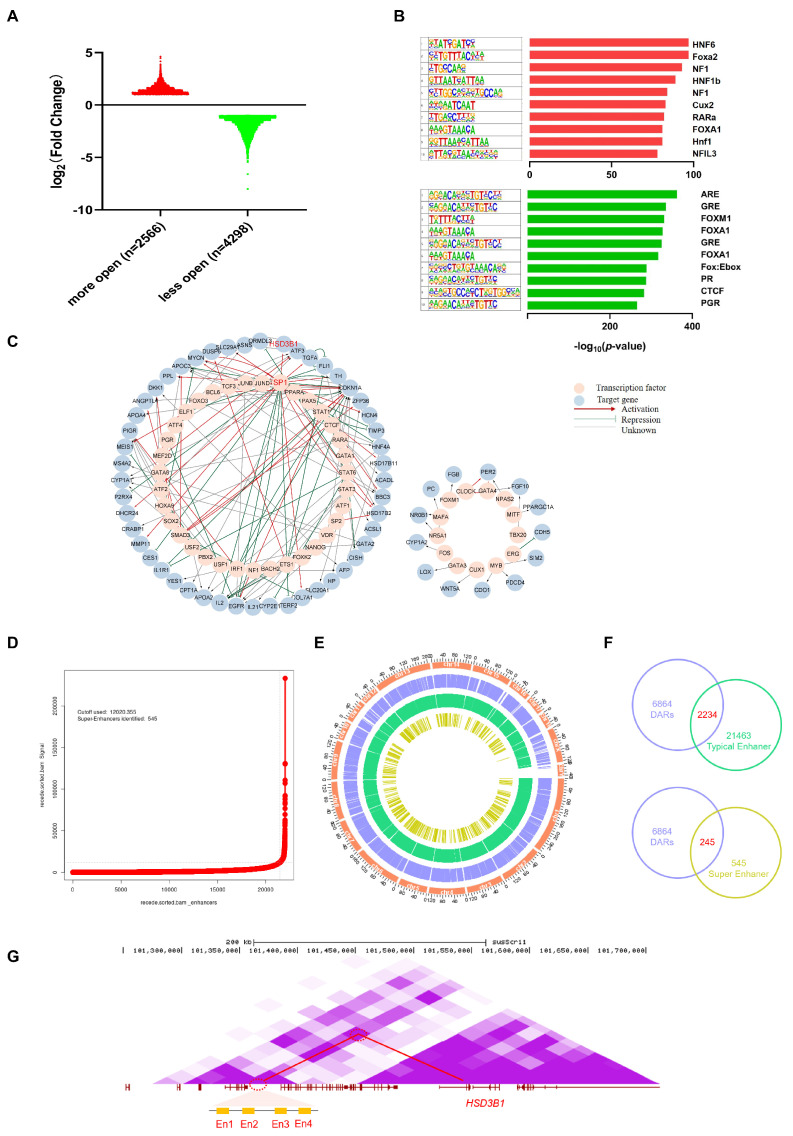
Identification of functional TFs and enhancer elements. (**A**) Bar graph and numbers show ATAC−seq peaks in more and less accessible regions, respectively. (**B**) Top 10 TF−binding motifs enriched in significantly increased (red) and decreased peaks (green). (**C**) TF regulatory networks of TF and target genes with differentially accessible peaks. (**D**) SE profiles and enhancer rank of H3K27ac peak categories defined by H3K27ac ChIP−seq signals. Cutoffs for distinguishing between Ens and SEs are shown as dashed lines. (**E**) Circos diagram represents the distribution of DARs, Ens, and SEs. Chromosomal information (orange), DARs (blue), Ens (green), and SEs (yellow) are successively shown from the outer to the inner circles. (**F**) Regions shared in DARs, Ens, and SEs. (**G**) The relative positions of four enhancers, including En1, En2, En3, En4 (yellow square), and the *HSD3B1* gene; purple shading for the Hi−C data represents loop intensity, and a highlighted Hi−C loop is delineated with red circles. The genome coordinate positions of En1, En2, En3, and En4 are chr4: 101,355,633−101,356,009, chr4: 101,385,387−101,385,990, chr4: 101,404,814−101,405,333, and chr4: 101,406,287−101,406,525, respectively.

Compared to the I group, 2566 more open accessible chromatin regions and 4298 less open regions were identified in the C group (Figure 6A). The analysis of accessible chromatin regions via ATAC-seq not only identifies regulatory regions for transcription but can also infer relevant TF within them [39,45,46]. Therefore, we identified a list of 310 known and 18 de novo motifs that were significantly enriched in upregulated peaks and 330 known and 21 de novo motifs in downregulated peaks, compared to the all-peak background (Supplementary Table S5 and S6). The top 10 TF binding motifs that were significantly enriched by increased peaks were the binding sites of *HNF6*, *Foxa2*, *NF1-halfsite*, *HNF1b*, *NF1*, *Cux2*, *RARa*, *FOXA1*, *Hnf1*, and *NFIL3*. The top 10 significantly enriched TF binding motifs by decreased peaks were the binding sites of *ARE*, *GRE*, *FOXM1*, *FOXA1*, *Fox:Ebox*, *PR*, *CTCF*, *PGR*, *Foxa2*, and *BORIS* (Figure 6B). Of these, certain motifs were enriched in both the upregulated and downregulated peaks, such as *FOXA1*, implying that they might function differently by targeting different genes.

### 3.6. Identification of Functional TFs and Enhancer Elements Located in Differential Accessible Chromatin Regions

TFs often function in a network using crosstalk to regulate gene transcription. To more accurately determine the TFs that play a regulatory role based on the chromatin open region, further study how TFs regulate downstream genes, and establish the potential connection of enriched TFs, we first developed an analytical framework that utilizes both our transcriptomic and chromatin accessibility data. From the ATAC-seq results, we obtained 310 known TF binding motifs in increased peaks and 330 known TF binding motifs in decreased peaks. Considering the motifs in the ROCs of C and I groups, we screened out 395 TFs. Based on the TRRUST database, we gathered our TF-target regulatory relationships, including 114 TFs and 272 target genes. Of these, 62 target genes were differentially expressed in our RNA-seq results, corresponding to 52 TFs and resulting in 123 TF-gene pairs (Supplementary Table S5). Of these, the mode of regulation (activation or repression) of the 65 regulatory relationships was unknown, and we first identified the mode of regulation according to our ATAC- and RNA-seq data. Interestingly, we identified *HSD3B1* gene, the dominant enzyme in phase I of androstenone metabolism [26], as one of the target genes of TF *SP1* (Figure 6C).

A hallmark of regulatory areas is chromatin accessibility, which is frequently required for enhancer and TF coordination. All identified peaks were primarily enriched in regions containing cis-regulatory elements, including introns and intergenic regions (Figure 2B). Chromatin accessibility is required for enhancer activity, and ATAC-seq is even more sensitive for the detection of active cis-regulatory elements than H3K27ac ChIP-seq in certain cases [47,48]. In order to improve the accuracy of enhancer recognition, we utilized previously published H3K27ac data of castrated pig livers from UC Davis FAANG and identified 545 super-enhancers (SEs) and 21,463 typical enhancers (Ens) using the ROSE software (http://younglab.wi.mit.edu/super_enhancer_code.html) [40,41] (Figure 6D, Supplementary Tables S7 and S8). The Circos plot shows the distribution profile of differentially accessible regions (DARs), Ens, and SEs in the genome (Figure 6E). We compared the DARs with Ens and SEs, and supporting the accurate annotation of the open chromatin region as a putative enhancer, 2234 Ens and 245 SEs were obtained (Figure 6F, Supplementary Tables S9 and S10). Four Ens were identified based on chromosome location and Hi-C loop interactions, ranging from 195 kb to 144 kb away from *HSD3B1* (Figure 6G); serendipitously, chromatin accessibility in the four enhancer regions was significantly upregulated in the C group. This finding may provide one explanation for the increased expression of *HSD3B1* following castration.

### 3.7. Screening of Molecular Markers for Androstenone-Related Enhancers

**Figure 7:**
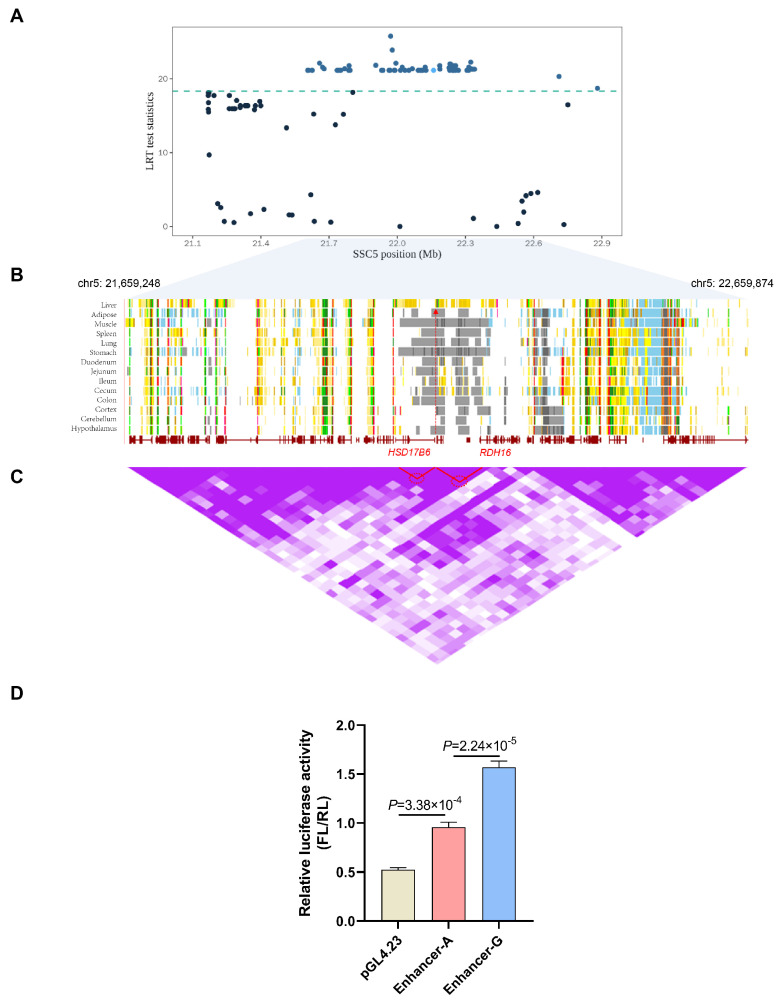
Screening of molecular markers for androstenone-related enhancers. (**A**) Manhattan plot displays the enrichment of signals associated with androstenone within the interest region. The SNP within the candidate enhancer region is highlighted with a light blue dot. (**B**) Chromatin states for different tissues in the interest region. Arrows in red indicate the liver-specific enhancer. (**C**) Hi−C data from UCSC browser. Purple shading for the Hi−C data represents loop intensity. Two highlighted Hi−C loops delineated with red circles are potential contacts between a candidate enhancer and *HSD17B6* and *RDH16*. (**D**) Luciferase reporter assay determined andEN activity with different genotypes (A and G).

To further characterize the androstenone-related enhancer region, we collected all pig androstenone-associated quantitative trait nucleotides (QTNs) from the ISWINE database [49] and then examined QTNs in the putative enhancer region. Using our pipeline, we characterized a putative enhancer region, and a significant SNP (rs334482774) located in this region is presented in Figure 7A. Moreover, data from Pan [50,51] annotated the candidate enhancer region as a liver-specific enhancer (Figure 7B). Based on the chromosomal position and Hi-C loop interaction (Figure 7C), the potential targets *HSD17B6* and *RDH16* were discovered, which have previously been reported as candidate genes for androstenone [25]. To evaluate the enhancer activity and effect of SNP rs334482774, we performed dual-luciferase reporter assays in HEK293T cells. The results confirmed the enhancer activity, and the G-allele of rs334482774 increased the activity of the enhancer (Figure 7D). Therefore, the SNP may be a candidate causal variant that regulates enhancer activity and ultimately affects boar taint.

## 4. Discussion

Boar taint, an unpleasant odor of meat, results from the excessive accumulation of androstenone in pig adipose tissue, rendering the meat unfit for human consumption [3,8], which could lead to economic losses. The effective reduction of boar taint can be achieved via castration [7,52], since it eliminates all testis-derived factors, including androstenone and all the sex hormones. Androstenone is synthesized in the testes and primarily metabolized in the liver, with any unmetabolized portion accumulating in the adipose tissue owing to its lipophilic properties [6,8]. Several studies have indicated an upregulation in the expression of genes associated with androstenone metabolism in the pig livers post-castration [11,12]; however, the underlying mechanism remains elusive. Hence, employing omics approaches to investigate the dynamic alterations in the liver of castrated and non-castrated pigs holistically can provide us with a comprehensive perspective.

The co-analysis of RNA- and ATAC-seq identified 227 overlapping genes, including the FMO family (*FMO5*) and the cytochrome P450 (*CYP1A2*, *CYP2A19*, and *CYP2E1*) and 17β-HSD families (*HSD17B11* and *HSD17B2*), which have previously been identified as candidate genes for boar taint [23,24]. The FMO family of enzymes converts lipophilic compounds into more polar metabolites [53], thus contributing to the decreased activity of lipophilic compounds; we observed a negative correlation between androstenone levels and the expression of *FMO5*, which is consistent with that seen in the liver of pigs with high and low androstenone levels. Studies have shown that androstenone can prevent *CYP2E1* induction by its substrate skatole in pig hepatocytes; skatole is another significant factor responsible for taint, apart from androstenone [54,55,56]. Moreover, *CYP2E1* expression was elevated in the castrated pigs; this implies that *CYP2E1* could also serve as an important marker of boar taint.

In addition to determining chromatin accessibility, ATAC-seq can be employed to track the activity of cis-regulatory elements [57]. In this study, we predicted TF binding motifs using the genome-wide analysis of chromatin accessibility. We also demonstrated the use of these data to correlate TF, open chromatin, and gene expression, thereby predicting the interactions of these regulatory elements to form a transcriptional network. In our TF regulatory networks, we identified the *HSD3B1* gene, which is a key enzyme in phase I of androstenone metabolism. However, little is known about its upstream regulators. Our results showed that TF-*SP1* not only acts as a potential regulator of *HSD3B1* but also targets 20 other genes, which are associated with androstenone metabolism [23]. In addition, as a hepatic reprogramming factor, the *HNF4A* gene can bind to chromatin to activate liver-specific gene expression [58], which may be an important target. Currently, limited research exists on TFs involved in androstenone metabolism, and most studies concentrate on the identification of important enzyme genes [22,23,24]. Therefore, another strategy is to focus on TFs. By integrating transcriptomic and chromatin accessibility data, we have expanded our understanding of the transcriptional mechanisms underlying gene expression in liver tissue. Furthermore, our findings suggest that chromatin accessibility and gene expression are not always positively correlated. Approximately 18% (41/227) of the overlapping genes showed opposite results, possibly due to the binding of transcriptional repressors to open chromatin regions [59].

An increasing quantity of epigenetic and transcriptional data have been provided by the FAANG project, which has contributed to the identification of regulatory regions in the porcine genome [50,51,60]. Although this type of data is useful for identifying regulatory regions, it is expensive and time-consuming. ATAC-seq may be applied to identify several of these elements because of its relatively low cost. Previous studies have highlighted the importance of non-coding region regulation [61,62,63], and enhancers located in non-coding regions have been extensively associated with transcriptional regulation related to hepatic function [64]. Numerous studies have identified hundreds of thousands of enhancer factors in liver tissue, encompassing various stages and breeds [65,66,67]; however, further exploration is needed for functional annotations. Here, we identified four enhancers of *HSD3B1* for the first time and observed a consistent enhancement in chromatin openness of these enhancers in the castrated group. We speculate that this may be one of the reasons why *HSD3B1* expression was elevated after castration.

Furthermore, genome-wide association studies have identified numerous molecular markers associated with pig production traits, and the investigation of mutations that directly affect gene expression has attracted significant interest. Because several SNPs are located outside the gene body, they have gone unnoticed, and SNPs found in intronic regions may also act as regulatory factors and potentially cause mutations [68,69,70]. Thus far, how genotype influences phenotype has been limited by only genomic sequence or gene expression profiles; thus, there is a current and pressing scientific need to characterize the variations in cis-regulatory elements.

An SNP associated with androstenone metabolism has been reported to be located in an intron [1], and subsequent ChIP-seq results showed that H3K27ac was also located in the corresponding region [67], indicating a probable reason for its cis-regulatory element. H3K27ac is a hallmark of Ens, and a cluster of large enhancers with a high density of H3K27ac is defined as SEs [40,41]. A 2013 study revealed that 64% of trait-associated non-coding SNPs occur within enhancer regions (covering approximately 33% of the genome) defined by H3K27ac, and a higher enrichment of trait-associated SNPs was observed in SEs than was observed in Ens [71]. Combining variations in noncoding regions, including enhancers and SEs, contributes to the elucidation of phenotypes. However, the number of androstenone-related SNPs is small due to the limited number of studies on androstenone. Here, andEN, with an androstenone quantitative trait nucleotide, was initially selected through multiple omics strategies; its association with androstenone metabolism needs more experimental verification, including the identification and verification of the target gene and the establishment of the association between the target gene and androstenone. With the discovery of additional SNPs, we believe that more useful SNPs will be identified, which will have significant implications for improving boar taint.

## 5. Conclusions

In this study, we systematically profiled chromatin accessibility and transcriptional dynamics in the liver tissue of castrated and intact full-sibling Yorkshire pigs and identified upstream TF *SP1* and four enhancers of *HSD3B1*. In addition, a candidate typical enhancer andEN with an androstenone quantitative trait nucleotide was identified. These findings present valuable information for understanding the regulatory mechanisms of androstenone metabolism and provide an important foundation for improving pork quality.

## Data Availability

The RNA-seq and ATAC-seq data presented in this study are openly available in National Genomics Data Center with the accession numbers PRJCA022590 and PRJCA022591.

## Supplementary Materials

The following supporting information can be downloaded at: https://www.mdpi.com/article/10.3390/biom14040427/s1, Table S1: qPCR primer sequence; Table S2: ATAC-seq data statistics; Table S3: RNA-seq data statistics; Table S4: Chromatin openness of differentially expressed genes; Table S5: TFs homer known motifs enriched in chromatin regions of liver; Table S6: TFs homer de novo motifs enriched in chromatin regions of liver; Table S7: Ens identified by H3K27ac data; Table S8: SEs identified by H3K27ac data; Table S9: DARs overlapped with Ens; and Table S10: DARs overlapped with SEs.
